# Invasive pneumococcal infections in Vellore, India: clinical characteristics and distribution of serotypes

**DOI:** 10.1186/1471-2334-13-532

**Published:** 2013-11-09

**Authors:** Viktor Molander, Camilla Elisson, Veeraraghavan Balaji, Erik Backhaus, James John, Rosemol Vargheese, Ranjith Jayaraman, Rune Andersson

**Affiliations:** 1 Institute of Biomedicine, Department of Infectious Diseases, Sahlgrenska Academy, University of Gothenburg, Guldhedsgatan 10 A, P.O. Box 7193, 40234, Gothenburg, Sweden; 2 Department of Microbiology, Christian Medical College, CMC & H, Ida Scudder Road Vellore-4, Vellore, India; 3 Department of Infectious Diseases, Skaraborg Regional Hospital, S-541 85, Skövde, Sweden

**Keywords:** Invasive pneumococcal disease, Pneumococcus, Vaccination, Pneumococcal vaccine, India, Serotype

## Abstract

**Background:**

*Streptococcus pneumoniae* infection is a serious problem worldwide and the case fatality rate remains high. The aim of this study was to analyze the distribution of pneumococcal serotypes causing invasive pneumococcal disease (IPD), to survey the potential coverage of present and future vaccines, and to investigate differences between serotypes and groups of serotypes with regard to manifestation, case fatality rate, age, and other risk factors.

**Methods:**

Isolates from 244 consecutive patients with IPD were collected at the Christian Medical College, Vellore, India between January 2007 and June 2011, and clinical data were obtained retrospectively. Clinical characteristics were analyzed both for individual serotypes and for those grouped as “invasive”, “pediatric”, or “vaccine” serotypes.

**Results:**

The serotype coverage for the pneumococcal conjugated vaccines (PCV) PCV7, PCV10, PCV13, PCV15, and pneumococcal polysaccharide vaccine (PPV) PPV23 was 29%, 53%, 64%, 66%, and 73%, respectively. The proportion of IPD caused by vaccine types was lower than pre-vaccination studies from other parts of the world. In adults, serotype 1 was mainly isolated from previously healthy patients without risk factors for IPD. This serotype caused more pneumonia and less meningitis than other serotypes, as was also noted for the “invasive” serotypes (1, 5, and 7 F).

**Conclusions:**

The most common pneumococcal serotypes in this study behaved in similar ways to those in countries where the PCV has been introduced. Also, the most common serotypes in this study are included in the new PCVs. Therefore, a national program of childhood immunization with PCV10/13 in India is likely to be successful.

## Background

*Streptococcus pneumoniae* infection is a serious problem worldwide [1,2]. It is a common cause of pneumonia, meningitis, and septicemia, and the case fatality rate remains high [3]. Globally, India has the highest number of deaths caused by pneumococcal infections among children below 5 years of age, partly because of its large population [2]. The incidence, severity, and mortality of the disease depend on host factors such as age, underlying disease, comorbid conditions, and immunosuppression, but also on the properties of the organism [4]. Pneumococci are divided into different serotypes based on various properties of the capsule, and 94 different serotypes have been identified so far. The serotypes have varying abilities to cause invasive disease, and some serotypes tend to infect certain risk groups, such as children or patients with underlying diseases [4-6]. The serotype distribution varies over time and with geographic location, and local surveillance is therefore needed to determine the regional spread [7].

Existing vaccines target the pneumococcal capsule and are serotype specific. The first immunogenic pneumococcal vaccine in children under the age of 2 years was the seven-valent pneumococcal conjugate vaccine (PCV7), launched in 2000. Today, three PCVs, which include 7, 10, and 13 serotypes, have reached the market, and a PCV15 is under clinical trial [8]. The introduction of PCV has led to a marked decline in the incidence of vaccine type-IPD in both vaccinated children and unvaccinated adults in several countries [9]. Unfortunately, an increase in the incidence of IPD caused by non-vaccine serotypes following vaccination is a concern, especially the emergence of a serotype 19A clone with a high case fatality rate and multi-drug resistance [10]. To be able to estimate the efficacy prior to starting immunization in an area, it is important to investigate the disease burden, risk factors for disease, and serotype distribution [7]. Thus, more studies in India regarding these topics are needed.

The aim of this study was to analyze the distribution of pneumococcal serotypes causing IPD, to survey the potential coverage of present and future PCVs, and to investigate differences between serotypes and groups of serotypes with regard to manifestation, case fatality rate, age, and other risk factors.

## Methods

### Study design

Invasive pneumococcal isolates were collected consecutively at the Christian Medical College and Hospital (CMC) in Vellore, India, between January 2007 and June 2011 as part of an international surveillance program. CMC Vellore is a tertiary care multispecialty hospital that caters to 2500 inpatients and 6000 outpatients every day. It serves patients from all over India as well as neighboring countries, although citizens of Tamil Nadu State are overrepresented. The total number of blood cultures performed in 2012 was 35,658. Isolates were serotyped using a co-agglutination test [11] and confirmed by PCR [12]. Clinical data such as risk factors, manifestation, and outcome were collected retrospectively from February to April 2012 from hospital records. All samples were taken as part of the standard clinical care and the data were obtained from the ordinary patient records without any additional tests or visits for the patients. Antimicrobial susceptibility was determined using agar dilution to obtain minimal inhibitory concentration (MIC) in accordance with CLSI guidelines [13,14]. The revised breakpoints from January 2008 were used (parenteral cut-offs). If several isolates were extracted from the same patient, only one isolate from the same disease episode was included in the study. The study design and proposals were approved by the ethics committee and Institutional review board (IRB) of Christian Medical College, Vellore, South India.

### Definitions

Invasive pneumococcal disease (IPD) was defined as isolation of pneumococci from normally sterile sites including blood, cerebral spinal fluid, pleural fluid, ascites, and synovial fluid. The local of infection was set by either positive cultivation or diagnosis by clinician. Case fatality was defined as death during the time of admission. Risk factors were defined as systemic autoimmune disease, cancer, cardiovascular disease, liver disease, kidney disease, lung disease, diabetes, HIV, other chronic conditions, splenectomy, previous skull fracture, immunosuppressive treatment, alcohol abuse, and smoking. Multi-drug resistance (MDR) was defined as resistant or intermediate resistant to ≥ 3 antimicrobial agents.

When analyzing individual serotypes, all patients were divided into children (0–17 years) and adults (≥ 18 years). The pneumococcal serotypes were divided into “invasive serotypes” (1, 5, 7(A-C + F)), “pediatric serotypes” (6(A-C), 9(A, L, N, V), 14, 19(A-C + F), 23(A-B + F)), “PCV10” (1, 4, 5, 6B, 7 F, 9 V, 14, 18C, 19 F, 23 F) and “PCV13” (PCV10 serotypes + 3, 6A, 19A) serotypes, similar to other authors [4,6,15].

### Statistical analysis

The data were analyzed using SPSS (IBM, version 20) and Microsoft Excel (version 14.1.4). Fisher’s exact test (two-sided) was used to calculate p-values, and p < 0.05 was regarded as significant.

## Results

Isolates from 244 patients with IPD were collected in this study. The ages ranged from 0–84 years, with a mean age of 29 years and median age of 28 years. There were 168 men and 76 women.

Risk factor data were available for 233 patients, of which 151 (62%) had at least one known risk factor for IPD. The most common comorbid conditions were chronic kidney disease (n = 37), cardiovascular disease (n = 31), malignant disease (n = 31), diabetes (n = 27), and chronic liver disease (n = 20). Other risk factors for pneumococcal disease such as skull fracture (n = 18) and immunosuppressive treatment (n = 32) were also present. Pneumonia (n = 102) was the most common clinical manifestation, followed by meningitis (n = 58), septicemia without known focus (n = 51), and peritonitis and arthritis (n = 21). Out of all patients, 216 (88%) were treated as inpatients and 22 (9%) were treated as outpatients (no data available for six patients, 3%). The duration of admission ranged from 1–48 days, with a median of 6 days. Altogether, 49 patients (20%) died during their hospital stay from causes related to the infection (25% in 2007, 18% in 2008, 31% in 2009, 18% in 2010, 16% in 2011).

**Figure 1:**
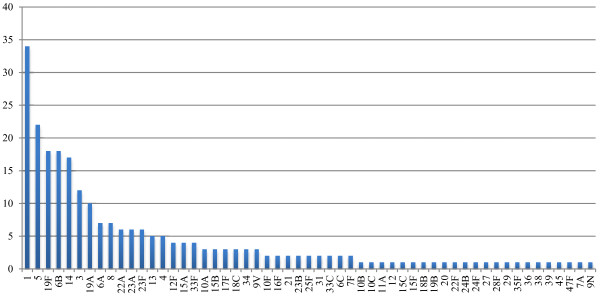
**Serotype distribution of *S pneumoniae *** **in samples collected between January 2007 and June 2011 at Christian Medical College and Hospital in Vellore, India (N = 244).**

Figure 1 shows the serotype distribution among all isolates. One of the isolates in serogroup 12 was not serotyped. Case fatality rate, risk factors, and clinical manifestations among patients infected by the five most common serotypes are shown in Table 1, and the same parameters related to groups of serotypes are shown in Table 2. The age distribution and the potential vaccine coverage in different age groups are shown in Table 3.

**Table 1:** Clinical characteristics of individual serotypes

**Serotype**	** All**	**Fatal outcome**		**Any risk factor**		**Pneumonia**		**Meningitis**	
	**No**	**No (%)**	**P**-**value**	**No (%)**	**P**-**value**	**No (%)**	**P**-**value**	**No (%)**	**P**-**value**
	**Children**	(**all** = **95**)		(**all** = **95**)		(**all** = **93**)		(**all** = **93**)	
*1*	*10*	0 (0%)	ns	2 (22%)	ns	7 (88%)	0.007	0 (0%)	ns
*5*	*6*	0 (0%)	ns	0 (0%)	ns (0.059)	2 (40%)	ns	3 (60%)	ns (0.084)
*19 F*	*11*	2 (18%)	ns	5 (46%)	ns	5 (46%)	ns	2 (18%)	ns
*6B*	*8*	2 (25%)	ns	4 (50%)	ns	2 (25%)	ns	3 (38%)	ns
*14*	*14*	0 (0%)	ns	5 (36%)	ns	4 (31%)	ns	3 (23%)	ns
**All serotypes**		12 (13%)		44 (46%)		38 (41%)		22 (24%)	
	**Adults**	(**all** = **138**)		(**all** = **138**)		(**all** = **136**)		(**all** = **136**)	
*1*	*24*	3 (13.0%)	ns	10 (43.5%)	<0.001	20 (87.0%)	<0.001	2 (8.7%)	0.038
*5*	*16*	4 (25.0%)	ns	11 (68.8%)	ns	9 (64.3%)	ns	2 (14.3%)	ns
*19 F*	*7*	2 (40.0%)	ns	6 (100%)	ns	3 (60.0%)	ns	2 (40.0%)	ns
*6B*	*10*	3 (30.0%)	ns	10 (100.0%)	ns	2 (20.0%)	ns	6 (60.0%)	0.022
*14*	*3*	1 (33.3%)	ns	2 (66.7%)	ns	3 (100.0%)	ns	0 (0%)	ns
**All serotypes**		37 (26.8%)		107 (77.5%)		64 (47.1%)		36 (26.5%)	

Clinical characteristics of the five most common pneumococcal serotypes in children and adults. P-values were calculated for each serotype compared with all other serotypes, using Fisher’s exact test (two-sided). ns = not significant.

**Table 2:** Clinical characteristics of grouped serotypes

**Serotype group**	**All patients**	**Fatal outcome**		**Any risk factor**		**Pneumonia**		**Meningitis**		<**2 years**	
		(**all** = **233**)		(**all** = **233**)		(**all** = **229**)		(**all** = **229**)		(**all** = **244**)	
	**No**	**No(%)**	**P**-**value**	**No(%)**	**P**-**value**	**No(%)**	**P**-**value**	**No(%)**	**P**-**value**	**No(%)**	**P**-**value**
*Invasive*	59	8 (14%)	ns	25 (46.3%)	0.002	40 (75.5%)	<0.001	8 (15%)	ns (0.07)	5 (9%)	ns (0.07)
*Pediatric*	91	20 (23%)	ns	53 (60.2%)	ns	30 (35.3%)	0.039	29 (34%)	0.03	26 (29%)	<0.001
*PCV10*	128	23 (19%)	ns	68 (55.7%)	0.003	65 (54.6%)	0.001	28 (24%)	ns	27 (21%)	ns
*PCV13*	157	32 (21%)	ns	87 (58.0%)	0.004	76 (51.7%)	0.004	31 (21%)	ns (0.06)	29 (19%)	ns
**All serotypes**	244	49 (21%)		151 (64.8%)		102 (44.5%)		58 (25%)		41 (17%)	

Clinical characteristics related to pneumococcal serotypes clustered into “invasive”, “pediatric”, *PCV*10 and *PCV*13 serotypes, with each group compared with all other serotypes. P-values calculated with Fisher’s exact test (two-sided).

**Table 3:** Vaccine coverage

	**Vaccines**	<**2 yrs**	**2**-**17 yrs**	**18**-**59 yrs**	≥**60 yrs**	**All ages**
	*PCV7*	23 (49%)	18 (36%)	19 (17.0%)	10 (29%)	70 (28.7%)
	*PCV10*	28 (60%)	29 (58%)	50 (44.6%)	21 (60%)	128 (52.5%)
	*PCV13*	32 (68%)	35 (70%)	63 (56.2%)	27 (77%)	157 (64.3%)
	PCV15*	33 (70%)	37 (74%)	64 (57.1%)	28 (80%)	162 (66.4%)
	PPV23**	33 (70%)	41 (82%)	75 (67.0%)	29 (83%)	178 (73.0%)
Total		*47*	*50*	*112*	*35*	*244*

Number (percent) of strains covered by *PCV*-7, -10, -13, the coming *PCV*15 and the *PPV*23 in different age groups and all ages. *N*=244 *PCV15 includes all *PCV*13 serotypes, 22 F and 33 F. **Pneumococcal polysaccharide vaccine 23, includes serotypes 1, 2, 3, 4, 5, 6B, 7 F, 8, 9 N, 9 V, 10A, 11A, 12 F, 14, 15B, 17 F, 18C, 19A, 19 F, 20, 22 F, 23 F, 33 F.

Among adults, serotype 1 was more significantly associated with lower frequency of predisposing factors than other serotypes. It also caused more pneumonia and less meningitis than all other serotypes in adults. Among children, serotype 1 caused more pneumonia than other serotypes. No significant differences were found in case fatality rates.

When clustering the serotypes as “invasive” and “pediatric”, some significant differences were observed (Table 2). “Invasive” serotypes were more often isolated from patients with no known risk factors for IPD than non- invasive serotypes. They were also more likely to cause pneumonia and less likely to cause meningitis. Children aged 0–1 were underrepresented in this group. The group of “pediatric” serotypes showed significantly lower ability to cause pneumonia and higher ability to cause meningitis than non-pediatric serotypes. This group was also significantly more prevalent in the 0–1 year-old age group. Serotypes included in the PCV10 and PCV13 vaccines were more often isolated from patients without risk factors for IPD and more likely to cause pneumonia, when comparing to all other serotypes.

Susceptibility to antimicrobial agents is shown in Table 4. When testing the antibiotic susceptibility, 11 (4.5%) of the isolates had reduced susceptible (intermediate or resistant, I or R) to penicillin. For erythromycin, 33 (13.6%) were non-susceptible. In total, 13 (5.3%) were MDR. The serotypes that included MDR isolates were 6B (5 isolates), 19 F (2), 1 (1), 14 (1), 19A (1), 23 F (1), 3 (1), and 6A (1). PCV13 covered all of the 13 MDR serotypes, while PCV10 covered 10 (77%) of the MDR serotypes. Serotypes 3 and 8 were significantly associated with higher susceptibility to any of the antimicrobial agents tested, while PCV10 and PCV 13 serotypes were both significantly associated with lower susceptibility (I or R) to any antimicrobial agent tested. We also present results excluding cotrimoxazole because of its widespread resistance (I + R = 83%), see Table 5.

**Table 4:** **Susceptibilities of *S. pneumoniae *** **isolates to antimicrobial agents. I** = **intermediate**, **R** = **resistant**

	**I**	**R**
*Penicillin* (*n* = *243*)	7 (2.9%)	4 (1.6%)
*Erythromycin* (*n* = *243*)	6 (2.5%)	27 (11.1%)
*Chloramphenicol* (*n* = *243*)	0 (0%)	4 (1.6%)
*Cotrimoxazole* (*n* = *243*)	32 (13.1%)	181 (74.2%)
*Cefotaxime* (*n* = *243*)	0 (0%)	1 (0.4%)
*Oxacillin* (*n* = *193*)	3 (1.2%)	8 (3.3%)
*Levofloxacin* (*n* = *182*)	3 (1.2%)	0 (0%)
*Linezolid* (*n* = *203*)	0 (0%)	0 (0%)
*Vancomycin* (*n* = *205*)	0 (0%)	0 (0%)
*Multidrug resistant* (*n* = *242*)		13 (5.3%)

**Table 5:** Susceptibilities of individual serotypes and grouped serotypes

			All antibiotics*		**Without cotrimoxazole**	
**Serotype**	**No. of strains**	**Missing**	**I**/**R**	**P**-**value**	**I**/**R**	**P**-**value**
*1*	34	8	25 (96.2%)	ns	2 (7.7%)	ns
*5*	22	6	16 (100%)	ns	1 (6.2%)	ns
*19 F*	18	6	12 (100%)	ns	6 (50%)	0.011
*6B*	18	5	13 (100%)	ns	5 (38.5%)	ns (0.069)
*14*	17	10	7 (100%)	ns	3 (42.9%)	ns
*3*	12	3	3 (33.3%)	<0.001	1 (11.1%)	ns
*19A*	10	2	8 (100%)	ns	3 (37.5%)	ns
*6A*	7	4	3 (100%)	ns	2 (66.7%)	ns (0.089)
*8*	7	0	1 (14.3%)	<0.001	0 (0%)	ns
*PCV10 types*	128	41	85 (97.7%)	<0.001	19 (21.8%)	ns
*PCV13 types*	157	50	99 (92.5%)	0.021	25 (23.4%)	0.033
**Total**	**244**	**83**	**142** (**88.2%**)		**30** (**18.6%**)	

Reduced susceptibility of serotypes to any of the antimicrobial agents tested, calculated both including and excluding cotrimoxazole. Each serotype/group was compared with all other serotypes. ns = not significant *Penicillin, erythromycin, chloramphenicol, cefotaxime, oxacillin, levofloxacin, linezolid, vancomycin.

## Discussion

The most common serotypes in the present study were 1, 5, 19 F, 6B, 14, and 3. This result is similar to a national Indian study from 1999, but with minor differences in order of prevalence, and a decreased prevalence of serogroup 7 [16]. A small study from the same area in India showed similar results [17]. A study of serotypes in South-East Asia showed a similar ranking order to our study [18]. In that review, analyzing 3067 isolates from 23 studies, the ranking order was 19 F, 23 F, 14, 6B, 1, and 3. In contrast, there are major differences when comparing these studies to those conducted in high-income countries where serotypes 9 V, 4, 7 F and 12 F are more common [4,7]. Longitudinal studies have shown that the incidence of serotype 1 varies over time, which might explain this observation [19,20].

The proportion of serotypes identified in the current study that are included in the vaccines PCV7, 10, and 13 for all ages was 29%, 53%, and 64%, respectively, and 54%, 66%, and 71%, respectively, for children <2 years . The vaccine coverage was higher among children <2 years, the age group that will be most in need of PCV. The overall coverage seems to be slightly lower than in many high income countries. In Sweden, the coverage for PCV7 serotypes prior to the introduction of PCV7 in the national immunization program was 42% among all ages and 62% among children 0–4 years of age [7]. When discussing this matter, one must consider the much higher prevalence of pneumococcal disease in India. Even if the vaccine coverage is lower than in other countries, the number of disease episodes and deaths that can be prevented with vaccination is substantial. However, because the percentages of isolates that are covered by the common conjugate vaccines are lower than they were in the U.S. prior to general vaccination, some of the herd effects that were observed in the U.S. might not occur to the same extent if general vaccination with PCV10 or PCV13 was started in India [21]. On the other hand, herd effects were observed in Europe despite a 15–20% lower coverage [22]. It is difficult to predict the rate of serotype replacement, which depends on several factors, such as the proportion of non-vaccine serotypes prior to vaccination, serotype coverage of the vaccine, and overcrowded living conditions.

When analyzing the properties of individual serotypes, clinically relevant findings were made. In adults, serotype 1 tended to infect patients without any known risk factor for IPD, and caused more pneumonia and less meningitis than other serotypes. This is consistent with other studies [5,7,23]. In children, less significant values were found. This can be explained by a lower number of isolates, but also highlights the fact that serotypes behave differently in children and adults. Only a few correlations were found when analyzing other individual serotypes, which is probably explained by too few isolates of each serotype. Analysis of the properties of individual serotypes should be interpreted carefully, as confounding factors might be present.

It should be noted that the properties of pneumococci are not only dependent on serotype. Genotypic differences within and between different serotypes are also highly important, and were not investigated in this study. It is noteworthy that serotype 1 infections in West Africa seem to have a completely different pattern, with high case fatality rates and a larger proportion of meningitis [24,25]. This might be explained by the fact that other clones of serotype 1 dominate in West Africa, as compared to Europe [26]. Although we did not perform any clonal analysis in this study, it is likely that isolates of serotype 1 from this study belong to the same clones that are found in Europe, because the clinical characteristics are very similar. Further studies of the clonality of Indian IPD isolates are needed.

When the serotypes were clustered, the group of “invasive” serotypes differed significantly from other serotypes, in similar manner to a previous Swedish study [6]. They tended to infect patients without any known risk factors for IPD, caused more pneumonia and less meningitis, and were also less common among children < 2 years of age. The group of “pediatric” serotypes also differed from other serotypes in terms of manifestation and age distribution. An international study, analyzing 796 isolates from adults from 10 countries, showed that host factors are of more importance than serotype when it comes to severity and outcome of pneumococcal disease [4]. Our present study showed no significant differences in case fatality rate between individual serotypes or when grouping them.

Only a small proportion of the isolates (n = 11, 4.9%) had reduced susceptibility to penicillin, and resistance to other antimicrobial substances was also low (except for cotrimoxazole). This is consistent with The Asian Network for Surveillance of Resistant Pathogens (ANSORP) data showing that India has the lowest incidence of penicillin-resistant *S. pneumoniae* in Asia [27]. Serotypes 3 and 8 were significantly associated with higher antimicrobial susceptibility than other serotypes. Also, serotypes included in PCV10 and PCV13 were significantly associated with higher antimicrobial resistance. All MDR isolates belonged to PCV-13 serotypes, whereas only 77% belonged to PCV-10 serotypes. Theoretically, widespread childhood vaccination with a vaccine protecting against a larger proportion of serotypes, which are often seen together with resistance, could have a positive impact on overall resistance in pneumococci. Resistance seemed to have no impact on outcome, but the number of patients was too small to permit any conclusion.

The relatively small number of patients presenting with IPD over the time period studied is explained by the fact that most patients in this district are relatively poor and cannot afford expensive investigations such as bacterial culturing. Also, prior antibiotic treatment (over the counter) before culture collection results in less verified IPD.

The gender distribution in this population is worth highlighting; in our study, 69% of IPD patients were men. This could be explained by that men are more likely to seek health care in India. Also, men are more exposed to socio-economically related risk factors, such as alcohol, smoking and labor in a polluted environment.

Children less than 1 year of age, an age group that usually has the highest incidence of IPD, were underrepresented in this study. In this population, children are often treated presumptively without investigation, as the cost of clinical testing (such as microbiological cultivation) is not affordable.

There are some limitations to the current study. Clinical data were collected retrospectively from medical records, which do not always accurately reflect the clinical situation. For example, smoking is not always mentioned in medical records, but we had to assume that a patient was a non-smoker if it was not mentioned. Also, as CMC is a renowned referral hospital and medical treatment is often costly, the selection of cases may not be representative of the population. Finally, the number of individual serotypes is quite small and analysis for each serotype was not possible. Therefore, clustering of the serotypes was performed.

Invasive pneumococcal infection is a serious problem worldwide, and India has the highest total number of children who die from pneumococcal disease [15]. The introduction of a PCV into the Indian national immunization program has been a matter of discussion for several years [28]. A recent randomized study from India shows that PCV10 is immunogenic and well tolerated when co-administrated with DTPw-HBV/HiB in Indian children [29]. PCV13 has also been proven to be immunogenic in other parts of the world [30].

## Conclusions

This study shows that serotype distribution in India remains stable. In addition, the distribution of pneumococcal serotypes and their clinical characteristics are similar to countries where the PCV has been introduced, which strengthens the hypothesis that introduction of a PCV in India might be as successful as it has been in high income countries. This study contributes to a further understanding of IPD in India, although more research is needed.

## Competing interests

Erik Backhaus is a member of the advisory board for Pfizer, Sweden, and a speaker at meetings sponsored by Pfizer.

## Authors’ contributions

VM and CE designed the study, collected the data, performed the statistical analysis, and wrote the manuscript. VB supervised the study and related work in Vellore. EB participated in the design of the study and assisted with writing the manuscript. JJ, RV, and RJ performed the serotyping. RA supervised the study, participated in the study design, and helped write the manuscript. All authors read and approved the final manuscript.

## Pre-publication history

The pre-publication history for this paper can be accessed here:

http://www.biomedcentral.com/1471-2334/13/532/prepub
